# Identification of a Potent and Broad-Spectrum Hepatitis C Virus Fusion Inhibitory Peptide from the E2 Stem Domain

**DOI:** 10.1038/srep25224

**Published:** 2016-04-28

**Authors:** Xiaojing Chi, Yuqiang Niu, Min Cheng, Xiuying Liu, Yetong Feng, Fuxiang Zheng, Jingjing Fan, Xiang Li, Qi Jin, Jin Zhong, Yi-Ping Li, Wei Yang

**Affiliations:** 1 MOH Key Laboratory of Systems Biology of Pathogens, Institute of Pathogen Biology, Chinese Academy of Medical Sciences & Peking Union Medical College, Beijing 100176, China; 2 Institute of Human Virology and Key Laboratory of Tropical Disease Control of Ministry of Education, Zhongshan School of Medicine, Sun Yat-sen University, Guangzhou 510080, China; 3 Institut Pasteur of Shanghai, Shanghai Institutes for Biological Sciences, Chinese Academy of Sciences, Shanghai 200031, China

## Abstract

Hepatitis C virus (HCV) envelope proteins E1 and E2 play an essential role in virus entry. However, the fusion mechanisms of HCV remain largely unclear, hampering the development of efficient fusion inhibitors. Here, we developed two cell-based membrane fusion models that allow for screening a peptide library covering the full-length E1 and E2 amino acid sequences. A peptide from the E2 stem domain, named E27, was found to possess the ability to block E1E2-mediated cell-cell fusion and inhibit cell entry of HCV pseudoparticles and infection of cell culture-derived HCV at nanomolar concentrations. E27 demonstrated broad-spectrum inhibition of the major genotypes 1 to 6. A time-of-addition experiment revealed that E27 predominantly functions in the late steps during HCV entry, without influencing the expression and localization of HCV co-receptors. Moreover, we demonstrated that E27 interfered with hetero-dimerization of ectopically expressed E1E2 in cells, and mutational analysis suggested that E27 might target a conserved region in E1. Taken together, our findings provide a novel candidate as well as a strategy for developing potent and broad-spectrum HCV fusion inhibitors, which may complement the current direct-acting antiviral medications for chronic hepatitis C, and shed light on the mechanism of HCV membrane fusion.

Since its initial identification in 1989, hepatitis C virus (HCV) has been found all over the world, with 7 distinct genotypes and 67 confirmed and 21 unassigned subtypes^1^. Approximately 3% of world’s population is infected, making HCV a serious global health problem^2^. Exacerbating the issue, there is currently no vaccine for HCV, and it is estimated that an additional 3–4 million new infections will occur each year^3^. Nonetheless, a large number of compounds have been successfully introduced by combining virological models with high-throughput screening approaches. Although the US Food and Drug Administration recently approved several direct-acting antivirals (DAAs), including Telaprevir, Boceprevir, Sofosbuvir and Viekira Pak, access to these medications is limited by their high cost. Moreover, certain subgroups of difficult-to-treat patients may require adjunctive therapeutic approaches^4^,^5^. In addition, the drugs that specifically target virus enzymes, such as protease inhibitors, frequently induce resistant mutations. Indeed, evidence shows that the current treatment regimens have resulted in the selection of drug resistant HCV variants^6^; therefore, novel drugs and new strategies are still urgently needed.

HCV is a small, enveloped single-strand RNA virus that belongs to the Hepacivirus genus in the Flaviviridae family. Cell entry by HCV is a multi-step process that begins with attachment of a viral particle to the cell surface via attachment factors, followed by a complex process involving a series of specific cellular entry co-receptors, including scavenger receptor class B type I (SR-BI)^7^, tetraspanin CD81^8^, claudin-1^9^ and occludin^10^,^11^ tight junction proteins. Receptor tyrosine kinases epidermal growth factor receptor, ephrin receptor A2^12^, Niemann-Pick C1-like 1 and iron uptake receptor transferrin receptor 1 are also suggested to play roles in HCV entry^13^,^14^. Envelope protein E1 and E2-mediated interaction of HCV with entry factors leads to internalization of the virus via clathrin-mediated endocytosis^15^, followed by fusion of the viral membrane with the early endosome membrane at low pH^16^. The development of HCV pseudotype particles (HCVpp)^16^,^17^ and infectious cell culture-produced particles (HCVcc)^18^,^19^ over the past decade has greatly advanced our understanding of the HCV lifecycle.

The crystal structure of the E2 core has recently been determined, revealing a compact architecture composed of a central immunoglobulin-fold β-sandwich flanked by two additional protein layers as well as many regions without regular secondary structure^20^,^21^. The data obtained indicate that the core E2 ectodomain lacks the structural hallmarks of fusion, suggesting that E1 alone or E1 associated with E2 might participate in fusion. However, the structural data available for E1 and E2 are too limited to explain the fusion mechanism; in particular, the cellular and viral factors involved in membrane fusion remain to be identified. In addition to transmission through circulating particles, HCV can transmit directly into neighboring cells, i.e., cell-to-cell transmission, which was first suggested after the observation of infected cell foci in infected human livers by RNA imaging analysis^22^ and recently confirmed using a similar approach^23^. Although several host entry factors have been implicated in this process, the viral determinants and molecular mechanisms involved in fusion need to be further characterized.

Accordingly, we report the discovery of E27, a 35-aa peptide from the E2 stem domain that potently inhibits HCV infection by blocking E1E2-mediated membrane fusion. Our findings reveal new insight into HCV fusion and will help in the development of novel antivirals.

## Results

### Identification of an E2-derived HCV fusion inhibitory peptide

**Figure 1:**
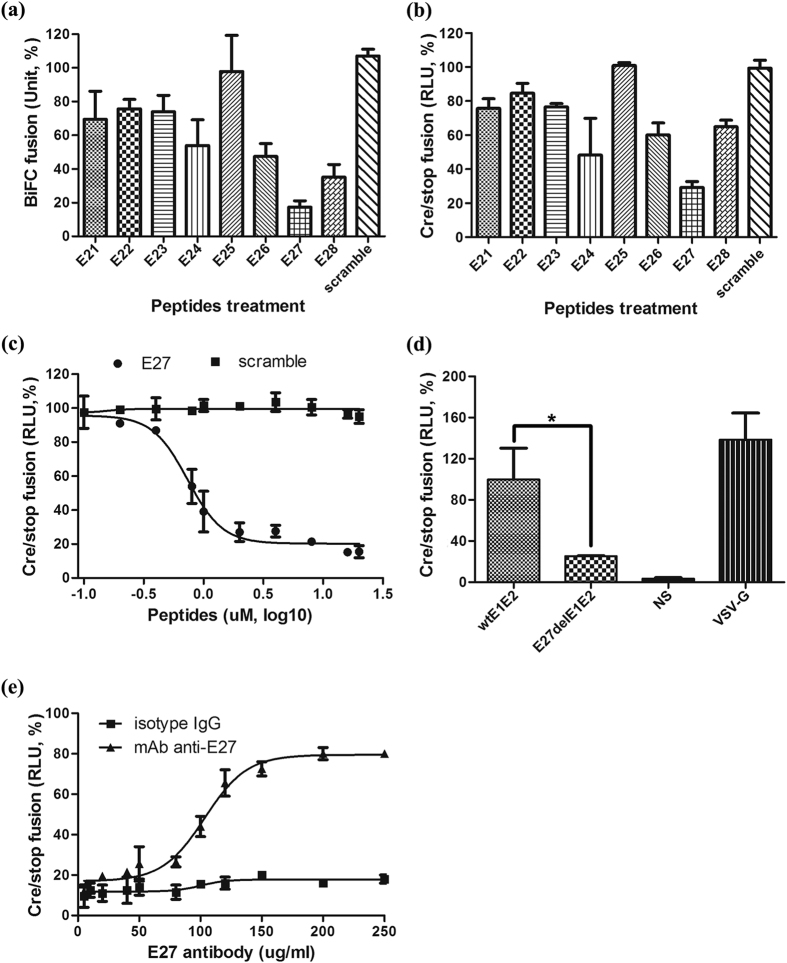
E27 inhibits HCV glycoprotein E1E2-mediated membrane fusion. **(a)** 293T cells co-expressing E1E2 and VN-CLDN1 were placed on top of Huh7.5.1 cells expressing VC-CLDN1. Membrane fusion was initiated by changing the culture medium to a low pH buffer, and the peptides were added at a final concentration of 5 μM. Fusion was measured at 12 h post-treatment by recording yellow fluorescent syncytia. **(b)** 293T cells co-expressing E1E2 and pCMV-Stop-Luc were placed on top of Huh7.5.1 cells expressing Cre recombinase. Membrane fusion was initiated by changing the culture medium to a low pH buffer, and the peptides were added at a final concentration of 5 μM. Membrane fusion was quantified at 12 h post-treatment with a luciferase assay. **(c)** Serially diluted E27 was added to the Cre/stop fusion system and triggered at low pH. Luciferase levels were measured after 24 h. A scramble peptide was included as a negative control. P = 0.03. **(d)** E1E2 mutant with the E27 region deleted was generated and named E27delE1E2. The membrane fusion properties of wtE1E2, E27delE1E2 and VSV-G were measured using the Cre/stop fusion system. NS (non-expressing envelope group) was included as a negative control. **(e)** A monoclonal antibody against E27 (mAb) was prepared and serially diluted for treatment in the Cre/stop fusion system. An isotype IgG from mouse was included as a negative control.

The membrane fusion process is a promising antiviral target for enveloped viruses, and low pH-dependent HCV membrane fusion, a critical step during virus entry, requires both viral envelope proteins and cellular factors. To identify fusion inhibitors and investigate the fusion mechanism, we devised two cell-based HCV fusion assays that can be quantified using either yellow fluorescent protein (bimolecular fluorescence complementation, the BiFC system, (see Supplementary Fig. S1a) or a luciferase reporter (the Cre/stop system, see Supplementary Fig. S1b) and then screened a library of 36 overlapping peptides (30-mers offset by 15 amino acids) covering the full-length E1E2 of HCV strain H77 (GenBank Accession No. NP_671491). Several peptides from the C-terminus of the E2 ectodomain (residues 641–715) efficiently inhibited fusion, whereas other peptides either failed to exert any effect or had only a marginal effect (see Supplementary Table S1). To further optimize the peptide length and sequence for enhancing fusion inhibition activity, we focused on and synthesized additional eight peptides (35-mer offset by 10 amino acids) covering E2 (611–715) (see Supplementary Table S2). We finally identified peptide E27 (671–705) as possessing the highest activity in both fusion assay systems (Fig. 1a,b), with an anti-fusion EC_50_ (half maximal effective concentration) value of 734.2 ± 9.3 nM (Fig. 1c).

To confirm the effect of the E27-containing region on the fusogenic property of E1E2, we constructed an E1E2 mutant with the E27 region deleted (named E27delE1E2) and evaluated its capacity in the Cre/stop fusion assay. As shown in Fig. 1d, compared with wild-type E1E2 and VSV-G, E27delE1E2-mediated fusion was significantly reduced, indicating that the E27-containing region is important for the fusion of the HCV envelope with target cells. Consistently, E27 efficiently suppressed wild-type E1E2-mediated fusion but not that of the E27delE1E2 mutant (see Supplementary Fig. S3).

To further characterize the importance of the E27-containing region in the fusion process, we raised a monoclonal antibody against peptide E27. Interestingly, this anti-E27 antibody, but not a control isotype IgG, can inhibit E1E2-mediated fusion (EC_50_ = 108.75 ± 8.4 μg/ml, Fig. 1e). These results demonstrate that the peptide E27 from the E2 stem domain plays a critical role in membrane fusion.

### E27 inhibits cell entry and infection of HCV genotypes 1–6

**Figure 2:**
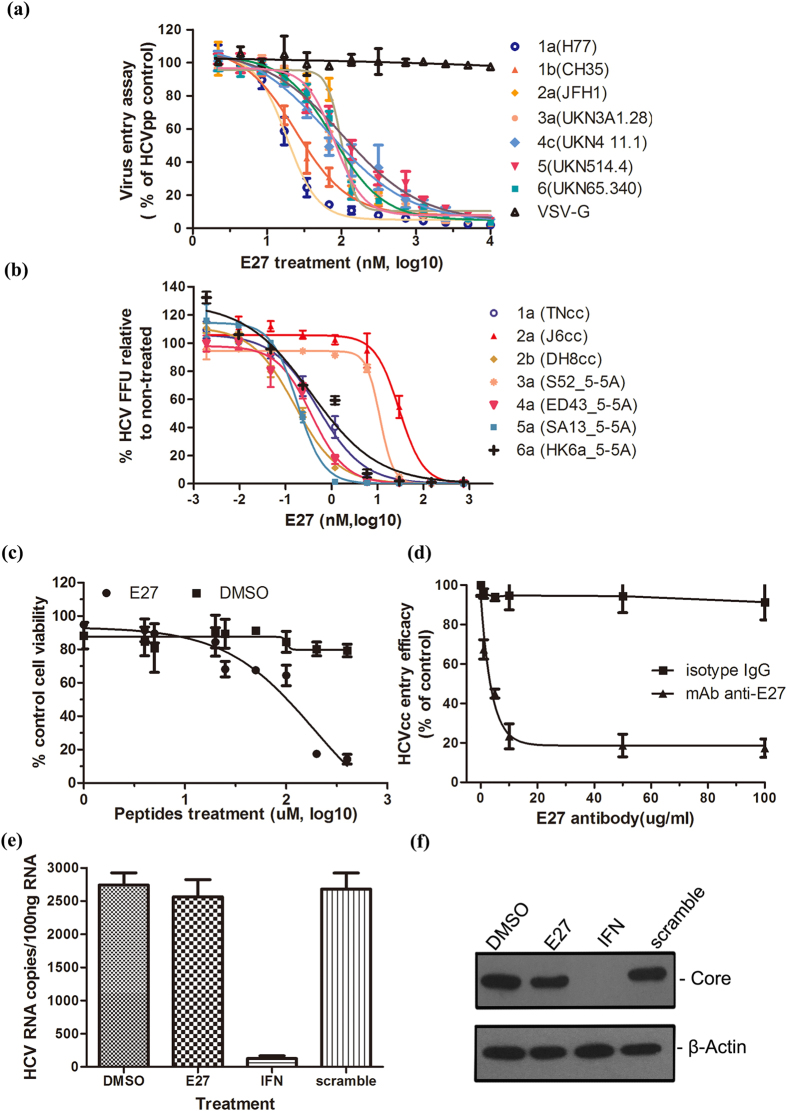
Broad-spectrum anti-HCV activity of E27. **(a)** VSV-Gpp (VSV-G-pseudotyped lentivirus) and HCVpp packaged with E1E2 from major genotypes were used to infect Huh7.5.1 cells in the presence of serially diluted E27 for an entry assay. The results were calculated relative to DMSO-treated cells. **(b)** E27 showed broad-spectrum antiviral activity against HCV full-length and 5’UTR-NS5A recombinants of genotype 1–6. Serially diluted E27 was mixed with first-passage virus stock and then incubated with Huh7.5.1 cells for 8 h before changing the medium. The number of FFU per well was determined at 48 h post-infection using a FFU assay. Values are the means of triplicate experiment ± SEM. **(c)** Cytotoxicity of E27 toward Huh7.5.1 cells by the MTT assay. **(d)** A neutralizing antibody against E27 was incubated with HCVccs (Jc1-luc) for 1 h and then added to Huh7.5.1 cells. Infection was evaluated after 72 h by determining luciferase activity. Isotype IgG served as the negative control. **(e**,**f)** 2^−^3^+^ replicon cells were treated with DMSO, E27 (1 μM), Interferon (IFN, 0.5 nM) or scramble peptide for 48 h. Intracellular HCV RNA was determined by qRT-PCR **(e)**, and the core protein was measured by Western blotting using the anti-HCV core antibody **(f)**. The experiment was performed in triplicate, and the error bars represent the standard error of the means (SEM).

To understand the potential determinants of E27 in HCV infection, we first analyzed the amino acid conservation of E27 sequences among HCV strains representative of seven major genotypes and subtypes, revealing strict conservation for >75% of the residues (see Supplementary Fig. S4a). The antiviral activity of E27 was further determined using both HCVpp packaged with E1E2 from genotypes 1–6 and infectious HCV cell culture systems, inducing full-length and 5’UTR-NS5A recombinants of genotypes 1–6^24^,^25^,^26^, and EC_50_ values were calculated from concentration response curves (Fig. 2a,b). For HCVpp assays, the EC_50_ of E27 ranged from 18.36 ± 0.77 nM to 104.8 ± 5.21 nM depending on the genotypic origin of the envelope protein (Fig. 2a). However, E27 did not inhibit the entry of VSV-G–pseudotyped lentivirus, suggesting the specificity of E27 inhibition (Fig. 2a). For HCVcc assays, the EC_50_ was determined to be 0.16 ± 0.03 nM to 29.48 ± 0.19 nM, lower than that determined using HCVpp (Fig. 2b). We further checked the cytotoxicity of the peptide and calculated a CC_50_ value for E27 of 180.3 μM in Huh7.5.1 cells, representing a therapeutic index (CC_50_/EC_50_) of 6,116–114,375 (Fig. 2c). Moreover, the monoclonal antibody against E27 (anti-E27) was demonstrated efficient HCV-neutralizing activity compared with the isotype control IgG (Fig. 2d). To eliminate the influence of E27 on HCV genomic RNA replication, a full-length HCV replicon model was used. Consistent with our expectation, E27 treatment had no impact on HCV RNA replication (Fig. 2e) or the intracellular HCV core protein level (Fig. 2f) in replicon cells. Taken together, peptide E27 appears to be a broad-spectrum HCV entry inhibitor with high potency.

### E27 targets the late steps of HCV cell entry

**Figure 3:**
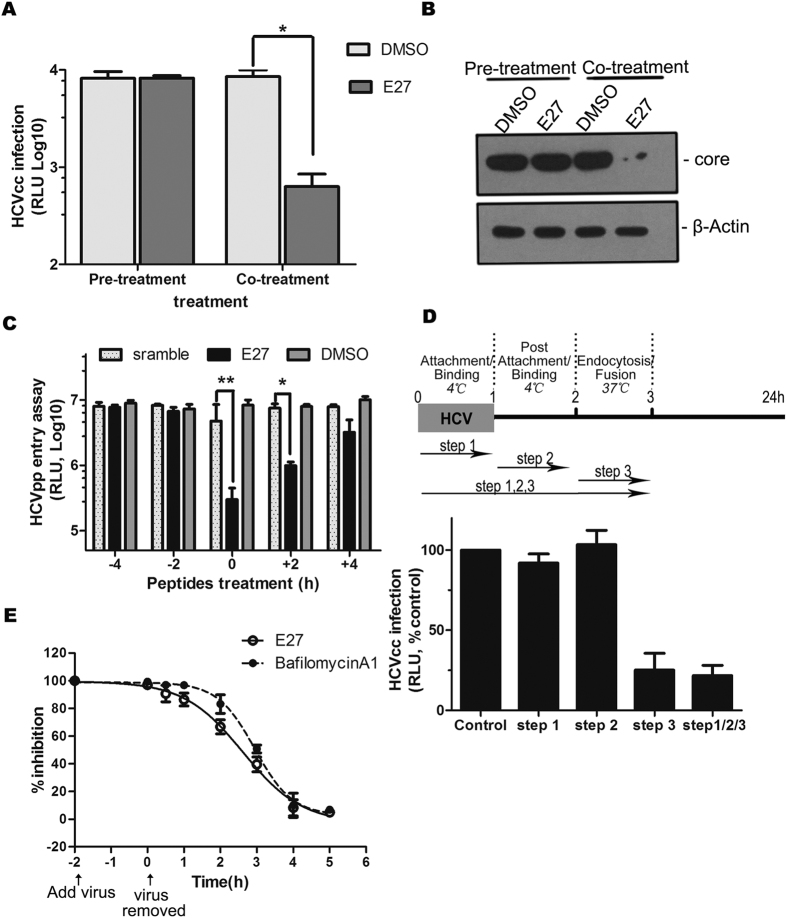
E27 inhibits HCV entry at late steps. **(A)** Group 1, virus was purified by ultracentrifugation at 28,000 × g for 4 h to remove the peptide and then used to infect Huh7.5.1 cells; Group 2, the peptide-virus mixture was directly added to the cells. After 48 h, the cells were lysed for luciferase assays. *P = 0.04. **(B)** Immunoblotting using the anti-HCV core antibody was performed according to the same experimental design described in Fig. 3A. (**C**) E27, scramble control peptide (1 μM), or DMSO (0.5%) was added to Huh7.5.1 cells 2 or 4 h prior to (−2 h and −4 h) or after (+2 h and +4 h) inoculation of HCVpp or together with the virus (0 h). For the −2, −4 h cells, the peptides were removed, whereas the 0, +2, and +4 h cells were incubated with the peptides along with the virus for a total of 12 h. The cells were lysed for luciferase activity at 48 h post-infection. **P = 0.007. **(D)** Step 1: 1 μM E27 were added to Huh7.5.1 cells during the HCV attachment/binding, and the virus and E27 were both removed after 1 h; Step 2: 1 μM E27 were added to cells during HCV post attachment/binding for 1 h at 4 °C and then removed before the cells were shifted to 37 °C; Step 3: 1 μM E27 were added to cells at the endocytosis/fusion step at 37 °C, the cells were washed after 1 h and incubated in 10%FBS medium; Step 1 2 3: E27 at 1 μM was incubated with cells in all the steps. **(E)** HCVcc (Jc1-luc) was added to Huh7.5.1 cells at 4 °C for 2 h. Unbound virus was removed, and the cells were shifted to 37 °C (0 h). E27 (1 μM) or bafilomycin A1 (10 nM) was added to the medium and incubated for 2 h prior to removal. The infected cells were incubated at 37 °C for 48 h prior to the luciferase assay. Inhibition was calculated as the % relative to infections with inhibitors when added at 5 h post-temperature shift (100%) and that with DMSO (0%). The fitted lines represent sigmoidal time-dependent curves (mean of n = 3; error bars, SD).

E27 was proven to be a fusion inhibitor and could inhibit HCV entry (Figs 1 and 2). To rule out the possibility that E27 directly acts on the integrity of HCV particles, concentrated JFH-1 HCVcc were treated with 1 μM E27 or DMSO as a control for 2 h at 37 °C and then loaded onto a sucrose cushion for ultracentrifugation to pellet viruses. After removal of the unbound peptide, the viruses were resuspended and used to infect naïve Huh7.5.1 cells. Compared with the significant suppression observed with co-treatment, pre-treatment of HCV with E27 showed no detectable effects on HCV infection (Fig. 3A,B), suggesting that E27 has no virocidal effect on HCV.

Next, to further corroborate that E27 targets HCV fusion, we sought to define the steps of HCV cell entry targeted by E27. It was observed that E27 exhibits the best inhibitory activity when added to cells together with the virus or within 2 h of infection, though no inhibition was observed when E27 was added prior to infection (Fig. 3C). In addition, we added E27 to Huh7.5.1 cells at different time intervals during the early phase of HCV infection. Three different time-points assays were conducted: cells were exposed to HCV for 1 h at 4 °C (attachment/binding period). Virus was removed, and the cells were washed with serum-free medium and incubated at 4 °C for another 1 h (postattachment/binding period). The cells were then washed and incubated for 1 h at 37 °C (endocytosis/fusion period). Finally, the cells were washed and incubated in 10% FBS-containing culture medium for 24 h, E27 was added at one of these steps. As shown in Fig. 3D, the addition of E27 during the late entry step led to a strong inhibition of HCV infection, whereas no inhibition was observed when E27 was added during the attachment or binding step. Moreover, we compared inhibitory kinetics between E27 and bafilomycin A1, a well-recognized inhibitor of fusion during virus infection. As shown in Fig. 3E, the inhibition curves were quite similar for E27 and bafilomycin A1. Altogether, these results support our previous results that E27 blocks the fusion step of HCV entry.

### E27 does not modulate the expression and localization of major HCV co-receptors

**Figure 4:**
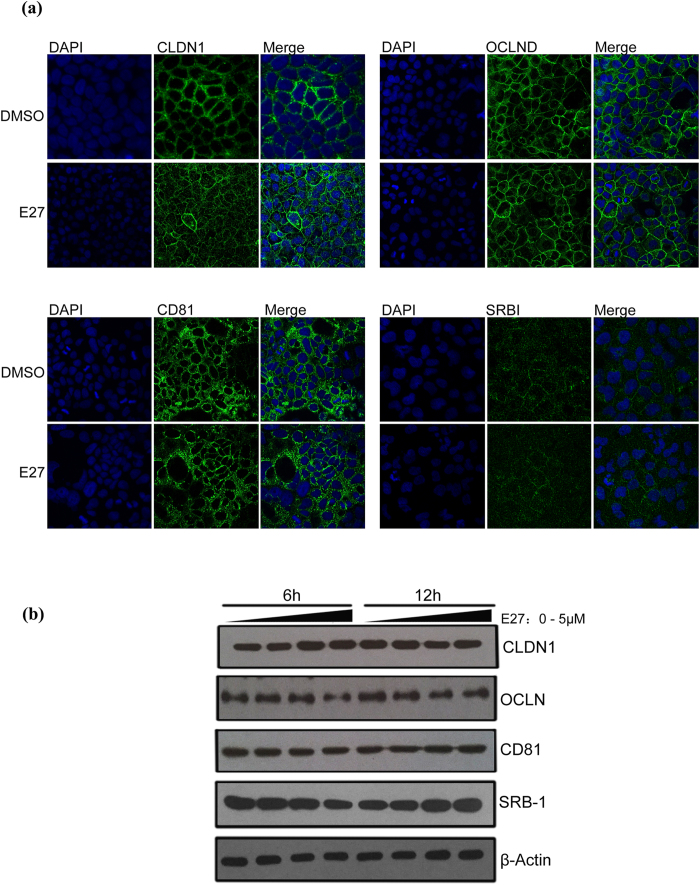
E27 affects neither HCV co-receptor localization nor expression levels. **(a)** DMSO and E27 (1 μM) were added to Huh7 cells and allowed to incubate for 24 h. CLDN1, OCLND, CD81 and SR-BI were stained with corresponding antibodies (green), and nuclei (blue) were stained with DAPI. **(b)** Huh7 cells were treated with 0, 0.1, 1 or 5 μM of E27, and the expression levels of CLDN1, OCLND, CD81 and SR-BI were analyzed by Western blotting. The expression of β-actin was also analyzed as a loading control.

HCV entry is a multistep process involving host factors, with CD81, SR-BI, claudin-1 and occludin being identified as essential entry co-receptors. Studies have suggested that some HCV inhibitors disrupt the integrity of tight junctions by inducing the rapid internalization of proteins^27^,^28^,^29^,^30^. Therefore, we examined whether E27 modulates the expression of the above mentioned cellular factors. Huh7 cells were treated with E27 for the indicated period, and entry factors were analyzed by immunofluorescent staining and Western blotting (WB). The results showed no detectable effect of E27 on the subcellular distribution and expression of these co-receptors (Fig. 4a,b). Therefore, at its antiviral dose, E27 is unlikely to inhibit HCV entry by interfering with co-receptor function.

### Mutations in E1 result in resistance to E27 treatment

**Figure 5:**
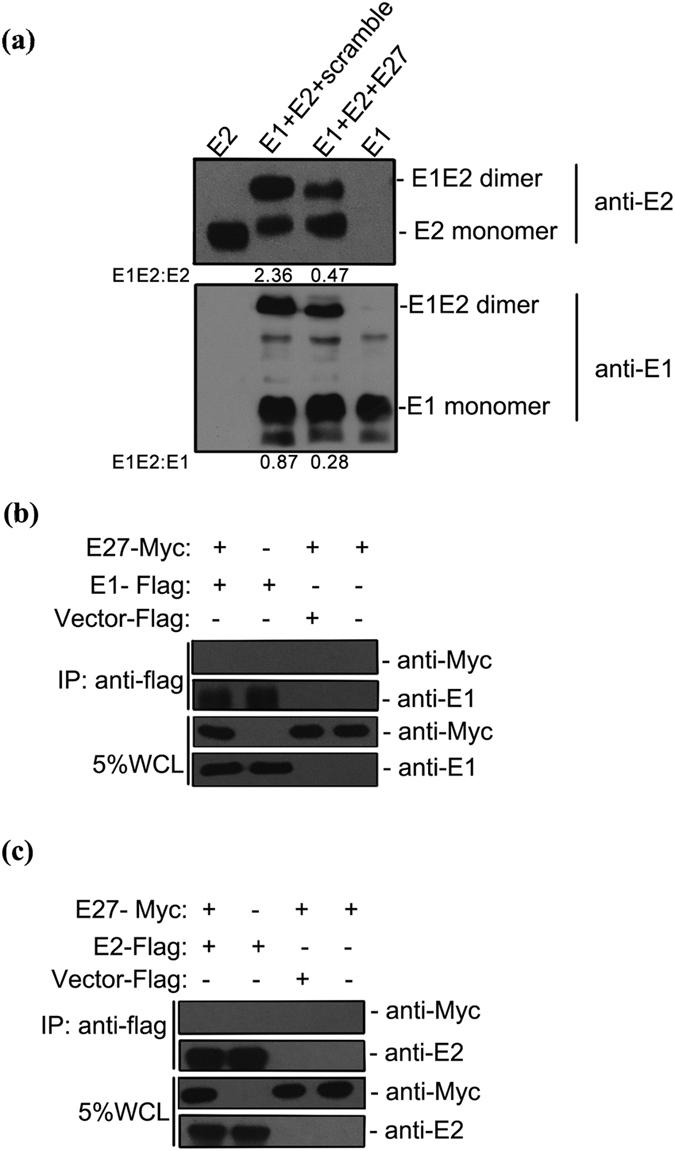
Effect of E27 on E1E2 heterodimerization. **(a)** Analysis of the E1E2 heterodimer by native-PAGE. A plasmid expressing the E27 peptide or a scramble control peptide was co-transfected into Huh7.5.1 cells with a plasmid encoding E1E2. The single E1 or E2 plasmid was used as the control. At 24 h post-transfection, cells were lysed for Western blotting using antibodies recognizing E1 or E2. **(b,c)** Interaction between the E27 and E1/E2 glycoproteins was determined by co-immunoprecipitation. A plasmid expressing Myc-tagged E27 was co-expressed with either Flag-tagged E1 (b) or Flag-tagged E2 (c). Cell lysates were immunoprecipitated with an anti-Flag antibody and detected with an anti-Myc antibody or an anti-E1- or E2 antibody. The empty Flag vector was used as a negative control.

Both E1 and E2 are required for HCV fusion: E1 and E2 assemble on the surface of the HCV particle as large covalent complexes stabilized by disulfide bonds, whereas they interact to form a non-covalent heterodimer in infected cells^31^,^32^. Very recently, it was reported that E1 forms a trimer on the HCV particle in an E2-dependent manner^33^. In contrast to E1, the recombinant E2 ectodomain has been suggested to be monomeric, with no homo-oligomeric form of E2 detected^20^,^21^. To ascertain the possible targets of E27, we first performed Western blotting to determine the effects of E27 on the dimerization of the E1 and E2 glycoproteins. Co-expression of E1, E2 and E27 in cells caused a significant decrease in E1E2 heterodimers (Fig. 5a). Nevertheless, co-immunoprecipitation assays showed that neither E1 nor E2 interact independently with E27 (Fig. 5b,c). Therefore, we speculate that E27 inhibition of HCV fusion may be achieved by disturbing the dimerization of E1 and E2.

**Figure 6:**
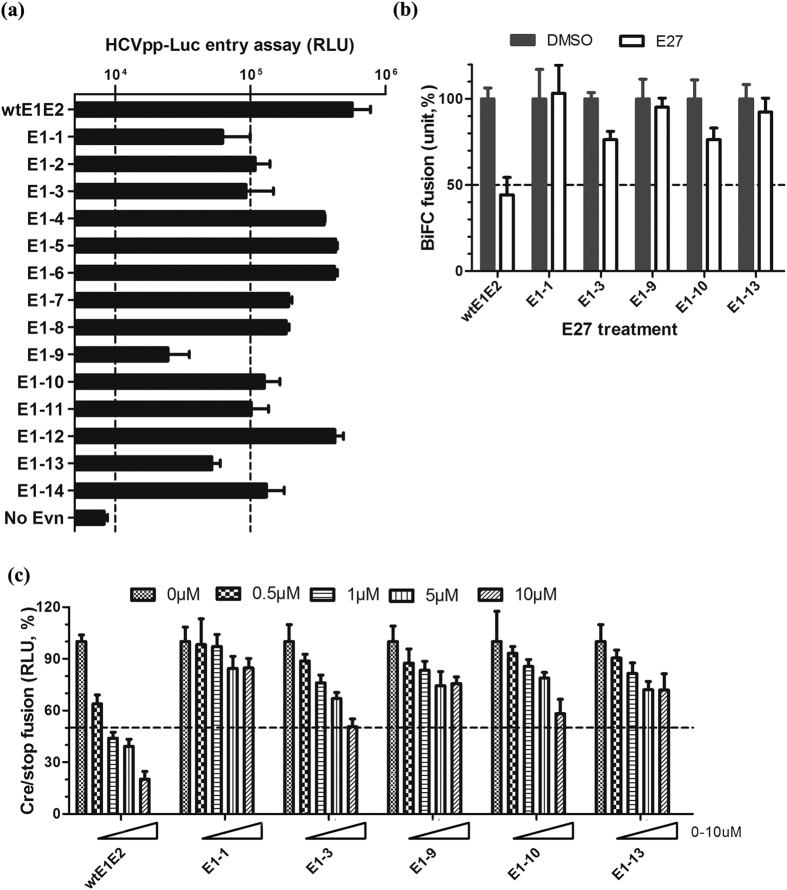
Mutations in E1 lead to resistance to E27. **(a)** HCVpp with mutated E1 was added to Huh7.5.1 cells in the presence of polybrene and HEPES. The cells were lysed at 48 hours post-spin-infection and assayed with the luciferase assay system. **(b)** Wild-type E1E2 (wtE1E1) or E1E2 carrying the indicated point mutations was used in the BiFC fusion system in the presence of 1 μM E27. Fusion was measured by recording yellow fluorescence syncytia. **(c)** Wild-type E1E2 or E1E2 carrying the indicated point mutations was used in the Cre/stop fusion system in the presence of 0, 1.25, 2.5, 5, 10 μM E27. Luciferase activity was measured after 12.

As E1 has been proposed to be a fusion protein that can interact with E2 through its transmembrane domain^34^, we hypothesized that E27 acts by targeting this domain. To examine the regions putatively targeted by E27, we first designed 14 mutated E1 variants (H77 strain) spanning amino acids 314–383 to map the region of E1 critical for fusion. Five residues were sequentially substituted by amino acids with opposite charges; hydrophilic amino acids were substituted with alanine and hydrophobic amino acids with glycine (summarized in Table S3). Our biochemical analysis indicated that E1E2 heterodimers for all variants were correctly expressed (data no shown). Using the HCVpp entry model, host cell entry was reduced more than 10-fold with the E1-1(314–318), E1-3(324–328), E1-9(354–358), E1-10(359–363) and E1-13(374–378) mutants (Fig. 6a). E1-13 was used as the control because it contains the E1E2 cleavage site mutation, which could lead to incorrect heterodimerization and inefficient fusion. Both the E1E2 wild-type and mutant constructs were employed in the cell-cell fusion systems to detect their susceptibility to E27. As shown in Fig. 6b,c, mutations of E1-1 and E1-9 markedly increased resistance to E27 in the fusion assay. Interestingly, the mutated region in E1-1 (314–316) is extremely conserved among HCV genotypes (see Supplementary Fig. S4b), and the E1-9 mutation affects the GxxxG motif that is suggested to be involved in E1E2 homo-oligomerization stability. In conclusion, E1 (314–318) and E1E2 dimerization are likely the targets of E27.

### Combination of E27 with small molecule DAA drugs

**Figure 7:**
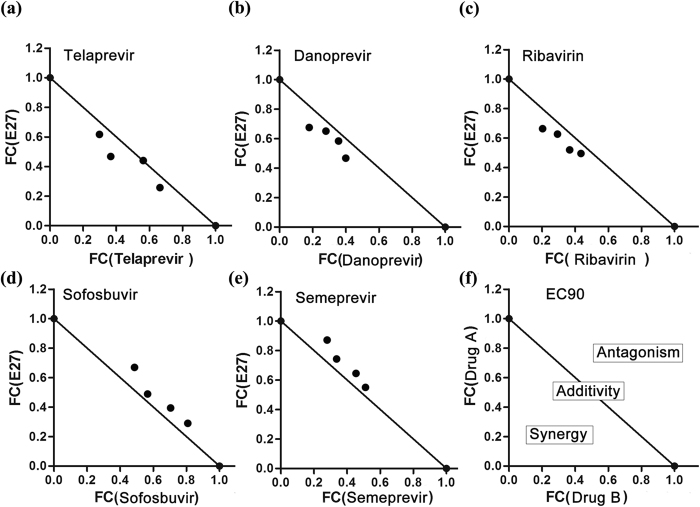
Analysis of the synergistic effect of the combination of E27 with Telaprevir, Danoprevir, Ribavirin, Sofosbuvir or Simeprevir. Huh7.5.1 cells were infected with HCVccs (Jc1-luc) and treated with combinations of E27 and Telaprevir, Danoprevir, Ribavirin, Sofosbuvir or Simeprevir. The cells were lysed for a luciferase assay after 48 h. The calculated EC_90_ values for the combinations were plotted as the fractional concentration (FC) of E27 and one of Telaprevir, Danoprevir, Ribavirin, Sofosbuvir or Simeprevir on the x and y axes, respectively. Synergy, antagonism and additivity are indicated in the graph at the right end of the lower graphs and are described in Materials and Methods.

Different from the mode of action of DAA drugs, E27 prevents HCV infection at the fusion step. To estimate the effects of drug combinations on the anti-HCV activity of E27, we examined its antiviral activity in combination with Ribvirin, Telaprevir (an NS3 protease inhibitor), Danoprevir (an NS3 protease inhibitor), Simeprevir (an NS3/4A protease inhibitor) and Sofosbuvir (an NS5B polymerase inhibitor). Huh7.5.1 cells were treated with E27 in combination with each DAA at various concentration ratios for 72 h, and the effect of each drug combination on HCV infection was analyzed using CalcuSyn software. An explanatory diagram of the isobologram is shown in Fig. 7, as described in Materials and Methods. As shown, all plots of the calculated EC_90_ values for E27 with the drugs are near the additive line. Additionally, we determined the degree of inhibition for each drug combination as the combination index (CI) at 50, 75 and 90% of the effective concentration using CalcuSyn. Table S4 provides an explanation. The results of combination tests indicate a slightly synergistic trend for Ribavirin and Telaprevir but slight antagonism for Simeprevir and Sofosbuvir to inhibit HCV infection in combination with E27.

### Optimization of E27 antiviral activity

**Figure 8:**
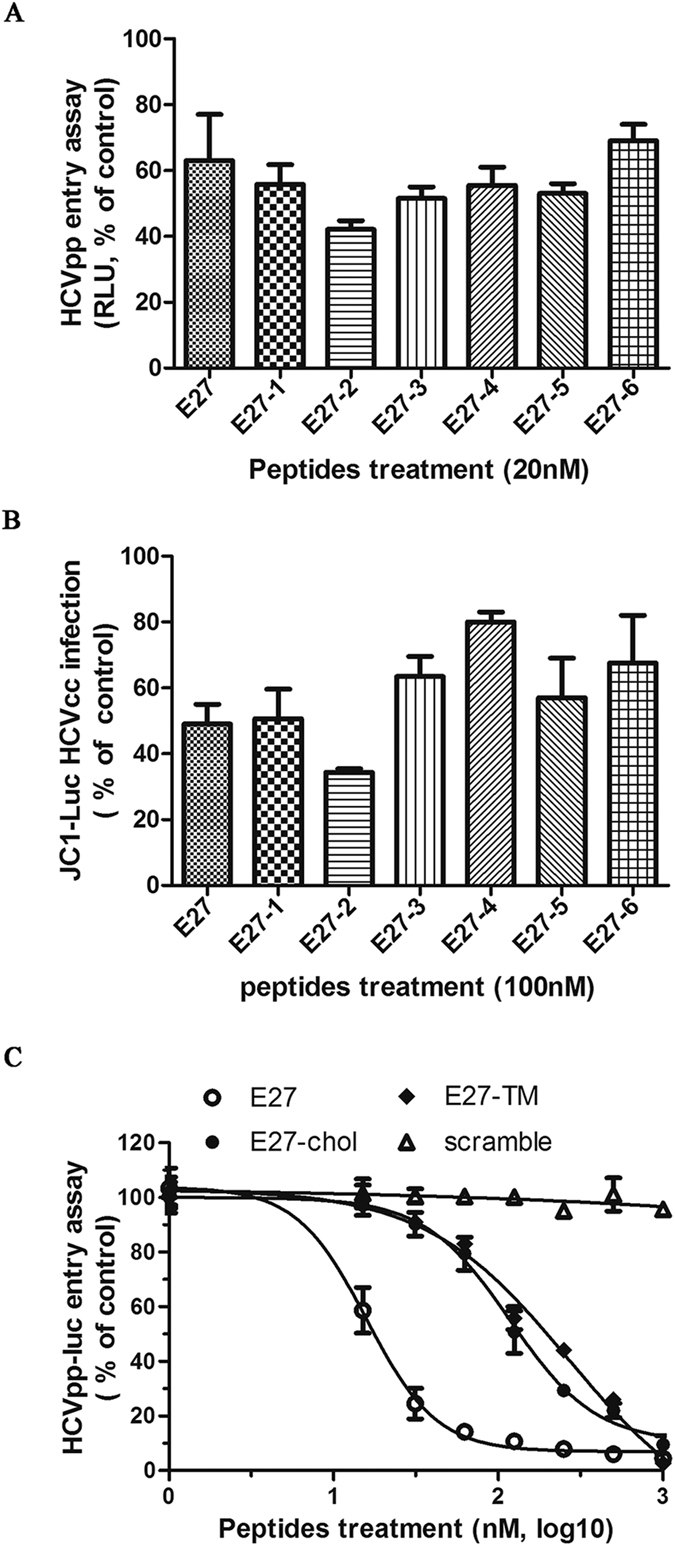
Optimization of E27 for anti-HCV activity. **(A,B)** Peptides were modified by removing 3 amino acids from the N- or C-terminus of the E27 parental peptide and tested for anti-HCV activity using the HCVpp system (a) or HCVcc system (b). The sequences of the peptides are summarized in Table S3. **(C)** E27 was modified by conjugating a cholesterol group or appending a short E2-transmembrane sequence to the N-terminus of the peptide to generate E27-chol or E27-TM, respectively. Anti-virus activity was measured by the HCVpp entry assay. E27 and a scramble peptide were included as controls.

Based on our findings, E27, composed of 35 amino acids, shows potent anti-HCV activity at low nanomolar or sub-nanomolar concentrations. We further optimized this peptide by altering the length by removing residues from the N- or C-terminus and found that shortening the peptide by 6 aa from the N-terminus (E27-2) slightly increased its antiviral activity compared to the parental peptide (Fig. 8A,B). The synthesis of highly hydrophobic peptides is inefficient, and E27 has a relatively high hydrophobicity and an undesirable thermal stability. Accordingly, we modified E27 by conjugating a cholesterol group or appending a short E2-transmembrane sequence to the N-terminus to generate two modified peptides designated E27-chol or E27-TM; however, significant improvements were not achieved (Fig. 8C). Overall, optimized peptide E27-2 showed the best inhibitory activity and economical efficiency.

## Discussion

Significant progress in the fields of hepatitis C pathogenesis and treatment has recently been made with in-depth basic and clinical research, yet the HCV membrane fusogenic mechanism remains poorly understood. Although increasing evidence suggests that E1 plays an important role in HCV fusion, its mechanism has not been established. In addition, the mechanism by which E2 participates in fusion is not clear. Contrary to previous speculation that HCV may be considered a class II enveloped virus, the crystal structure of the E2 core shows minimal homology to other members of the Flaviviridae family. Therefore, elucidation of the HCV fusion mechanism is hampered by a lack of a precise hypothesis and model. As demonstrated by HIV fusion studies, the discovery of an HCV fusion inhibitory peptide can provide an ideal tool for resolving this issue. After screening and optimization, we were able to identify a potent and broad-spectrum HCV fusion inhibitor, E27. Based on our data, we propose that the interaction between E1 and E2 might be an ideal target for designing HCV fusion inhibitors, as mutations in a specific region of E1 result in resistance to E27 and because E27 interfered with E1E2 dimerization. Finally, better activity and lower cost was achieved by shortening and optimizing this fusion inhibitory peptide from 35 aa to 29 aa.

Viral fusion proteins mediate host cell entry of enveloped viruses by merging the viral lipid envelope and cell membrane. In general, fusion proteins such as HIV gp41 and influenza hemagglutinin (HA) are considered to serve as a passive α-helical anchor to the virus envelope during its fusion with the cell membrane, and synthetic peptides derived from fusion proteins have been shown to inhibit entry of the corresponding virus^35^,^36^. However, the structure of the HCV E2 core domain has a compact, globular domain-like structure, with several β-strands and only two small α-helices, that does not undergo significant conformational or oligomeric rearrangements upon exposure to low pH^20^. Inhibition of membrane fusion by the E27 peptide identified in this study could be explained by two possibilities.

First, E27 may prevent fusion by interfering with E1 and E2 heterodimerization. This novel mechanism is distinct from other fusion inhibitors, such as the small molecules Flunarizine and pimozide, which were previously reported to inhibit HCV fusion by targeting a hypothetical fusion peptide in E1 (246–290)^37^. Resistance mutation E1-1 affects four amino acids, 312 to 315, in the cytoplasmic region of the E1 protein that are important for interaction with the core protein, and resistance mutation E1-9 maps to the E1 GxxxG motif, which plays an important role in E1E2 heterodimerization. Very recently, a study showed that E2-dependent E1 trimerization plays a central role in coordinating the entry function of the HCV glycoprotein and that the GxxxG motif is the main determinant of E1 trimerization. It has also been reported that the E1 trimer is mainly observed on HCVcc, whereas it is detected as a monomer within infected cells^33^, suggesting a structural transition between E1E2 heterodimers and trimers. Moreover, there is evidence that E1E2 cross-talk is important for conformational changes leading to membrane fusion^32^. Our Western blotting results also showed that E27 could decrease E1E2 heterodimer formation (Fig. 5a). Therefore, we can hypothesize that by targeting E1, E27 likely functions during the structural transition that might be a transient and intermediate state; the absence of this domain would impair HCV membrane fusion.

Second, it is possible that E27 induces E1E2 conformational changes. Although sequence analysis showed that peptide E27 (derived from E2 amino acids 671–705) is not strictly conserved, the residues at variable positions exhibit similar physicochemical properties, indicating the structural conservation of E27 among various HCV genotypes. In addition, Yao and colleagues recently showed that the parainfluenza virus 5 (PIV5) fusion protein adopts lipid-dependent conformations that can alter the conformation of the fusion protein from predominantly α-helical to β-strand, suggesting a viral-fusion model in which the fusion protein actively promotes membrane topological changes during fusion, with the β-strand as the fusogenic conformation^38^. Thus, it cannot be excluded that E27 is indeed the fusion peptide harbored in the E2 protein. Despite the fact that the E2 core structure was recently resolved, the crystal structure of the E27-containing region of the E2 protein is not yet available^20^. Theoretically, an E27 pairing region should be present in either E1 or E2 to fuse the viral envelop and cell membrane. Therefore, characterization of the functional and structural features of an E27-interacting partner will be the priority of further research.

HCV resistant variants against DAAs exist naturally and emerge frequently after virological response failure with suboptimal treatment, especially for protease inhibitors and first generation NS5A inhibitors. Therefore, novel anti-HCV targets and candidate inhibitors are still in need. In this study, the identification of a potent and broad-spectrum HCV fusion inhibitory peptide adds a new tool for developing novel antiviral drugs that target HCV entry, and this candidate peptide may contribute to DAA resistance in the future. In addition, the design approach used in this study may offer a widely applicable strategy for inhibiting viral infections. Importantly, we will be able to utilize this novel fusion inhibitor as a tool to dissect the molecular mechanisms of HCV fusion.

## Materials and Methods

### Cells and reagents

Human hepatocyte Huh7.5.1 was provided by Dr. Francis Chisari (Scripps Research Institute, CA, USA). HCV full-length genotype 1b replicon-containing cell line (2^−^3^+^) was provided by Dr. Stanley Lemon (University of Texas Medical Branch, TX, USA). HEK293T cells were obtained from ATCC. All cell lines were maintained in DMEM supplemented with Penicillin and Streptomycin, 1% NEAA, and 10% fetal bovine serum (Gibco, Carlsbad, CA, USA). Antibodies were obtained from Dr. Jane McKeating (E2, clone 3/11); Dr. Harry Greenberg (anti-E1, A4); Zymed (anti-CLDN1, anti-OCLN and anti-SRB-1); BD Biosciences (anti-CD81, JS-81); and Sigma (anti-Flag M2 and β-actin). Secondary antibodies are purchased from Santa Cruz (Santa Cruz, CA, USA), Jackson ImmunoResearch Laboratories (West Grove, PA, USA), and Molecular Probes (Invitrogen) (Carlsbad, CA, USA). Bafilomycin A1 were purchased from Sigma (St Louis, MO, USA). PEG-IFNα2b was from Schering Plough (Kenilworth, NJ, USA).

### Production of HCV Pseudoviral Particles and Cell Culture-Grown HCV (HCVcc)

To produce HCV pseudoviral particles (HCVpp) or VSV-G-pseudotyped lentivirus (VSV-Gpp), HEK293T cells were seeded one day before transfection at 2.5 × 10^6^ cells in a 10 cm plate in 10 ml of DMEM containing 10% FBS. The next day cells were transfected using PEI. The transfecting DNA mixture (1 ml) was composed of 15 μg of pNL-4.3-Luc-E-R-, either 15 μg of HCV E1E2 expression plasmid (genotypes 1a, 3a, 4c, 5 and 6 from Dr. F. Cosset^17^, INSERM, France, genotype 1b CH35 from Dr. Linqi Zhang)^39^, or 3 μg of pHEF-VSV-G (expressing VSV-G Env protein). Culture supernatants containing HCVpp or VSV-Gpp were collected at 48, 60 and 72 hours post-transfection and filtered through 0.22 μm syringe filter^40^. In our study, non-enveloped lentivirus particles (Bald virus) were also made as the negative control. Production procedure of HCVcc expressing firefly luciferase was described elsewhere^40^,^41^.

### Fusion assays

Inhibition of HCV envelope protein-mediated cell fusion by peptides was determined as previously described^42^. 293T “donor” cells were seeded in six-well plates (8 × 10^5^ cells/per well) and transfected with an HCV E1E2 expression plasmid; cells transfected with the pCMV-VSV expression plasmid were used for the positive control. For the Cre/stop fusion system, the cells were co-transfected with the pCMV-Stop-Luc plasmid (Addgene, MA, USA), and for the BiFC (bimolecular fluorescence complementation system) fusion system, cells were co-transfected with VN-CLDN1; cells co-transfected with the pCMV-Stop-Luc or Cre plasmid (or co-transfected with VN-CLDN1 or VC-CLDN1) were used as the positive control, respectively. Transfections were deemed successful when a control well transfected with a plasmid expressing a fluorescent reporter displayed a rate of transfection of ∼70%. Cells were incubated for another 12 h before they were dissociated from the wells using a non-enzymatic cell dissociation solution following the manufacturer’s instructions (Sigma), suspended in DMEM containing 10% FBS, and mixed with target cells.

Huh7.5.1 “target” cells were seeded in a 24-well plate at 1.5 × 10^5^ cells/well and grown for ∼24 h at 37 °C. The cells were transfected with the Cre plasmid for the Cre/stop fusion system or VC-CLDN1 for the BiFC fusion system. After 12 h, “donor” cells were placed on top of the “target” cells (5 × 10^5^ “donor” cells/well in 24-well plates) and co-cultured for 4 to 6 h at 37 °C. To initiate fusion, the medium was removed and replaced with fusion buffer (135 mM NaCl, 15 mM sodium citrate, 10 mM morpholineethanesulfonic acid, 5 mM HEPES, 1 mM EDTA [made fresh from a 10× stock and adjusted to the desired pH between 4.8 and 7.0 using HCl]) for ≤1 min. The low-pH buffer was replaced with DMEM containing 10% FBS and different concentrations of the peptide. After incubating the cells for 12 h at 37 °C, the cells were examined by fluorescence microscopy using filters with emission spectra of 510 to 560 nm. Green multinucleated cells were counted in each well.

### Peptides

The overlapping peptide library was designed based on the protein sequences of HCV glycoprotein E2 for HCV entry inhibitor screening. Each peptide in the library contains 35 amino acids with 15 amino acids overlapped by neighboring peptides. The peptides were synthesized with L-amino acids for at least 95% purity by Genscript (YourBio-Tech Partner, SH). All peptides were initially dissolved in DMSO at concentration of 5mM as stocks for library screening. In all experiments, we kept the amount of DMSO under 0.5% (v/v) expect when higher concentrations of peptides were needed (DMSO was 1% in those cases).

### HCV infection assay

Preparation of HCV HCVpp was performed as previously described^35^. For the virus entry assay, Huh7.5.1 cells were seeded in a 48-well plate at a density of 5 × 10^4^/well the day before infection. The next day, 200 μl of supernatant containing HCVpp, VSV-Gpp or bald viruses was added to each well in the presence of 8 μg/ml of polybrene and 2 μl of 2 M HEPES (pH 7.55) and spin infected for 1.5 h in a table-top centrifuge (2500 rpm, 30 °C) followed by another 1.5 h incubation in a CO_2_ incubator. The cells were lysed at 48 h post-spin-infection and assayed with Luciferase Assay System (Promega, Madison, WI, USA). All experiments were performed in triplicate. We typically obtained counts ranging from 10,000–900,000, whereas the background signal from the bald virus-infected sample is usually below 100.

The preparation of full-length (genotypes 1a, 2a, and 2b) and 5’UTR-NS5A (3a, 4a, 5a, and 6a) recombinant HCV of genotypes 1–6 was described previously^24^,^25^,^43^. The plasmids of these HCV recombinants were kindly provided by Dr. Jens Bukh (University of Copenhagen and Hvidovre hospital, Denmark). The E1 and E2 region of passage-recovered viruses were confirmed by sequence analysis. For virus infection assay, Huh7.5.1 cells were seeded in a 96-well plate at a density of 6 × 10^3^/well the day before infection. The infection was performed in a MOI of 0.1 FFU/cells in triplicate. After 48h of infection, the cells were fixed and FFU was determined under light microscope. The dose-response curves of HCVcc to E27 were plotted and EC_50_ of E27 was calculated accordingly using GraphPad Prism 5.

### Cytotoxicity assay

Huh7.5.1 cells were treated with E27 for the indicated concentration and period of time. After removal of E27, cells were incubated for an additional 48 h in 24-well plates. Cytotoxicity was determined using the MTT Cell Viability Assay Kit from R&D Systems (Minneapolis, MN, USA).

### Time-of-Addition Assays

Huh7.5.1 cells were seeded at 8 × 10^4^ cells/well in 24-well plates. The next day, virus inoculum was added to the plates, which were then incubated at 4 °C for 1 h. Unbound virus was removed by 3 washes with cold medium. The cells were then shifted to 37 °C and incubated. The specified inhibitors were added at specific time points during infection and were either removed by washing after two hours or added back as described in the Figure legends. Firefly luciferase activity was quantified at 48 h post-infection. The experiments were performed in triplicate.

### In-Cell Western (ICW)

Huh7.5.1 cells were seeded in 96-well plate format at 1 × 104 per well one day before HCVcc infection (MOI = 0.01). Twenty-four hours post virus infection, the cells were treated with peptides or various control for indicated time. Seventy-two hours post infection, the cells were fixed in the original wells with paraformaldehyde and permeablized with Triton X-100, followed with immunostaining with anti-HCV core antibody (1:400 dilution) and IRDye Secondary Antibody (1:1000 dilution, Li-Cor, Nebraska, USA). Images were obtained on Odyssey Infrared Imaging System (Li-Cor, Lincoln, NE, USA).

### Immunofluorescent Staining and Confocal Microscopy

Immunostaining of Huh7 cells was performed using antibodies against CLDN1, OClD, SR-BI or CD81 as previously descriped^11^,^40^. Images were captured on a Leica SPW5 confocal microscope. Nucleus was stained with DAPI.

### Western Blotting and Immunoprecipitation

For cell lysate preparation, monolayer cells were lysed on ice with lysis buffer supplemented with protease inhibitor cocktail (Sigma or Pierce). After electrophoresis, the separated proteins were transferred onto a nitrocellulose membrane (Bio-Rad, Hercules, CA), blocked with 10% milk for 1 h, and incubated with the primary antibody overnight at 4 °C. The secondary antibody used was a 1:2000 dilution of HRP-linked anti-IgG. The ECL reagent (Amersham Biosciences, Piscataway, NJ) was used as the substrate for detection. For immunoprecipitation-coupled Western blotting, cleared cell lysates were immunoprecipitated with anti-FLAG M2 affinity resin (Sigma) and washed with lysis buffer. Eluted samples were separated by 12–15% SDS-PAGE and immunoblotted with the indicated antibodies.

### Drug combination

The effects of drug combinations were evaluated using Huh7.5.1 cells and the computer software CalcuSyn (Biosoft, Cambridge, United Kingdom). Dose inhibition curves of two different drugs were plotted together; for each drug combination, the EC_90_ values of several combinations of two different drugs were plotted as the fractional concentration (FC) of both drugs on the x and y axes. An explanatory diagram of the isobologram is shown in the right side of the lower panels in Fig. 3. Combination indexes (CIs) were calculated at the EC_50_, EC_75_, and EC_90_ values using CalcuSyn. A CI value of less than 0.9 indicates synergy, a CI value ranging from 0.9 to 1.1 indicates additivity, and a CI value of more than 1.1 indicates antagonism. The explanatory diagram is shown in Table S4.

## Additional Information

**How to cite this article**: Chi, X. *et al*. Identification of a Potent and Broad-Spectrum Hepatitis C Virus Fusion Inhibitory Peptide from the E2 Stem Domain. *Sci. Rep*. **6**, 25224; doi: 10.1038/srep25224 (2016).

## Supplementary Material

Supplementary Information
