# Supplementary material for: Identification of a Potent and Broad-Spectrum Hepatitis C Virus Fusion Inhibitory Peptide from the E2 Stem Domain
Source: Sci Rep. 2016 Apr 28;6:25224. doi: 10.1038/srep25224 (PMC4848495; doi:10.1038/srep25224)

Supporting Information

Identification of a Potent and Broad-Spectrum Hepatitis C Virus Fusion Inhibitory Peptide from the E2 Stem Domain

Xiaojing Chi[1], Yuqiang Niu[1], Min Cheng[1], Xiuying Liu[1], Yetong Feng[2], Fuxiang Zheng[2], Jingjing Fan[1], Xiang Li[1], Qi Jin[1], Jin Zhong[3], Yi-Ping Li[2], Wei Yang[1]*

Supporting Tables and Figures

Supporting Table S1. Relative inhibition of HCV E1E2-mediated fusion by peptides.

Name	Sequence	% of fusion
HCV-1	YEVRNVSGVYHVTNDCSNASIVYEAADMIM	56.4
HCV-2	CSNASIVYEAADMIMHTPGCVPCVRENNSS	80.8
HCV-3	HTPGCVPCVRENNSSRCWVALTPTLAARNA	64.3
HCV-4	RCWVALTPTLAARNASVPTTTIRRHVDLLV	85.2
HCV-5	SVPTTTIRRHVDLLVGAAALCSAMYVGDLC	84.7
HCV-6	GAAALCSAMYVGDLCGSVFLVAQLFTFSPR	81.5
HCV-7	GSVFLVAQLFTFSPRRHETVQDCNCSIYPG	85.5
HCV-8	RHETVQDCNCSIYPGHVTGHRMAWDMMMNW	76.9
HCV-9	HVTGHRMAWDMMMNWSPTAALVVSQLLRIP	56.9
HCV-10	SPTAALVVSQLLRIPQAVVDMVAGAHWGVL	78.8
HCV-11	QAVVDMVAGAHWGVLAGLAYYSMVGNWAKV	121.6
HCV-12	AGLAYYSMVGNWAKVLIVMLLFAGVDGGTY	122.8
HCV-13	LIVMLLFAGVDGGTYVTGGTMAKNTLGITS	60.1
HCV-14	VTGGTMAKNTLGITSLFSPGSSQKIQLVNT	69.6
HCV-15	LFSPGSSQKIQLVNTNGSWHINRTALNCND	56.0
HCV-16	NGSWHINRTALNCNDSLNTGFLAALFYVHK	64.2
HCV-17	SLNTGFLAALFYVHKFNSSGCPERMASCSP	66.7

HCV-18	FNSSGCPERMASCSPIDAFAQGWGPITYNE	70.8
HCV-19	IDAFAQGWGPITYNESHSSDQRPYCWHYAP	129.8
HCV-20	SHSSDQRPYCWHYAPRPCGIVPAAQVCGPV	65.8
HCV-21	RPCGIVPAAQVCGPVYCFTPSPVVVGTTDR	63.7
HCV-22	YCFTPSPVVVGTTDRFGVPTYSWGENETDV	79.0
HCV-23	FGVPTYSWGENETDVLLLNNTRPPQGNWFG	145.0
HCV-24	LLLNNTRPPQGNWFGCTWMNSTGFTKTCGG	114.5
HCV-25	CTWMNSTGFTKTCGGPPCNIGGIGNKTLTC	87.4
HCV-26	PPCNIGGIGNKTLTCPTDCFRKHPEATYTK	121.6
HCV-27	PTDCFRKHPEATYTKCGSGPWLTPRCLVHY	65.1
HCV-28	CGSGPWLTPRCLVHYPYRLWHYPCTVNFTI	82.2
HCV-29	PYRLWHYPCTVNFTIFKVRMYVGGVEHRLE	83.2
HCV-30	FKVRMYVGGVEHRLEAACNWTRGERCNLED	99.6
HCV-31	AACNWTRGERCNLEDRDRSELSPLLLSTTE	56.9
HCV-32	RDRSELSPLLLSTTEWQVLPCSFTTLPALS	11.2
HCV-33	WQVLPCSFTTLPALSTGLIHLHQNVVDVQY	9.7
HCV-34	TGLIHLHQNVVDVQYLYGIGSAVVSFAIKW	24.9
HCV-35	LYGIGSAVVSFAIKWEYVLLLFLLLADARV	40.0
HCV-36	EYVLLLFLLLADARVCACLWMMLLIAQAEA	10.7

Each peptide from the library was used to treat Cre/stop fusion system at a final concentration of 1 μM. Luciferase reporter gene expression was measured for each treatment and divided by the solvent control (0.5% DMSO) as described in *Materials and Methods*.

Supporting Table S2. Sequences and anti-fusion activity of optimized peptides.

**Name**	**Sequence**	**EC_{50} μM**
E21	YPYRLWHYPCTINYTIFKVRMYVGGVEHRLEAACN	>20
E22	TINYTIFKVRMYVGGVEHRLEAACNWTRGERCDLQ	>20
E23	MYVGGVEHRLEAACNWTRGERCDLQDRDRSELSPL	>20
E24	EAACNWTRGERCDLQDRDRSELSPLLLSTTQWQVL	9.77±1.15
E25	RCDLQDRDRSELSPLLLSTTQWQVLPCSFTTLPAL	17.65±2.36
E26	ELSPLLLSTTQWQVLPCSFTTLPALSTGLIHLHQN	12.38±0.94
E27	QWQVLPCSFTTLPALSTGLIHLHQNIVDVQYLYGV	0.73±0.068
E28	TLPALSTGLIHLHQNIVDVQYLYGVGSSIASWAIK	>20

Supporting Table S3. Summary of the mutations in E1.

Name	Wild-type Seq	Mutated Seq	Position
**E1-1**	TGHRM	AAAAG	314-318
**E1-2**	AWDMM	GGAGG	319-323
**E1-3**	MNWSP	GAGAG	324-328
**E1-4**	TAALV	AGGGG	329-333
**E1-5**	VAQLL	GGAAA	334-338
**E1-6**	RIPQA	AGGAG	339-343
**E1-7**	IMDMI	GGAGG	344-348
**E1-8**	AGAHW	GAGAG	349-353
**E1-9**	GVLAG	AGGGA	354-358
**E1-10**	IAYFS	GGGGA	359-363
**E1-11**	MVGNW	GGAAG	364-368
**E1-12**	AKVLV	GAGGG	369-373
**E1-13**	VLLLF	GGGGG	374-378
**E1-14**	AGVDA	GAGAG	379-383

Supporting Table S4. Combination index (CI):

	EC_{50}	EC_{75}	EC_{90}
**E27:Telaprevir**	1.02851	0.94941	0.89352
**E27:Danoprevir**	0.93030	0.91808	0.91585
**E27:Ribavirin**	0.97973	0.92928	0.88903
**E27:Sofosbuvir**	1.23084	1.15136	1.09403
**E27:Simeprevir**	1.22522	1.17951	1.18414

Supporting Table S5. Optimization of the E27 sequence and antiviral activity

Name	Sequence	Length (aa)	Description
**E27**	QWQVLPCSFTTLPALSTGLIHLHQNIVDVQYLYGV	35	1-35
**E27-1**	VLPCSFTTLPALSTGLIHLHQNIVDVQYLYGV	32	4-35
**E27-2**	CSFTTLPALSTGLIHLHQNIVDVQYLYGV	29	7-35
**E27-3**	TTLPALSTGLIHLHQNIVDVQYLYGV	26	10-35
**E27-4**	QWQVLPCSFTTLPALSTGLIHLHQNIVDVQYL	32	1-32
**E27-5**	QWQVLPCSFTTLPALSTGLIHLHQNIVDV	29	1-29
**E27-6**	QWQVLPCSFTTLPALSTGLIHLHQNI	26	1-26

Supporting Figure legends

Supporting Figure S1. Cell-cell fusion assay.

(a) 293T cells expressing HCV E1E2 as the "donor cell" were transfected with Vn-CLDN1; 293T cells (top) or Huh7.5.1 cells (bottom) as the "target cell" were transfected with Vc-CLDN1. After 12 hours, the 293T cells were placed on top of the Huh7.5.1 cells, and the fusion buffer was added to initiate fusion. Green syncytia were observed following fusion mediated by HCV E1 and E2and VSV-G at low pH.

(b) 293T cells expressing HCV E1E2 as the "donor cell" were transfected with pCMV-stop-luc; Huh7.5.1 cells as the "target cell" were transfected with Cre plasmids. The donor cells were placed on top of the target cells after 12 h, and fusion was initiated by the addition of fusion buffer. Following fusion mediated by HCV E1 and E2 and VSV-G at low pH, the cells were lysed for a luciferase assay after 24 h.

Supporting Figure S2. Anti-HCV activity of HCV E1E2 glycoprotein peptides.

An overlapping peptide library was designed and synthesized based on the protein sequences of HCV (H77) E1 and E2 glycoproteins. HCVpp (H77) was packaged in 293T cells and used in the initial screening. Peptides (10 μM) were premixed with HCVpp and then added to Huh7.5.1 cells. At 48 h post-infection, the cells were lysed for luciferase activity measurement. The results are calculated as relative entry to the counts obtained from scrambled peptide-treated cells.

Supporting Figure S3. Deletion of the E27 region blocks E1E2 fusion.

The E27 region was deleted from the E1E2 plasmid, and a cell-cell fusion assay was performed according to the same experimental design described in Figure S1A.

Supporting Figure S4. Sequence analysis of E27, E1-1 and E1-9.

The amino acids of E27 **(a)**, E1-1 and E1-9 **(b)** are numbered with respect to the HCV polyprotein of the H77 infectious clone (accession number 870 AF009606, top row).

The degree of amino acid conservation at each position can be inferred from the extent of variability (with the observed amino acids listed in decreasing order of frequency from top to bottom) together with the similarity index according to the CLUSTAL W convention (asterisk, invariant; colon, highly similar; dot, similar).

Supporting Figure S5. The schematic diagrams show peptides, GxxxG and cleavage site of E1E2.

Supporting Figures

Supporting Figure S1.

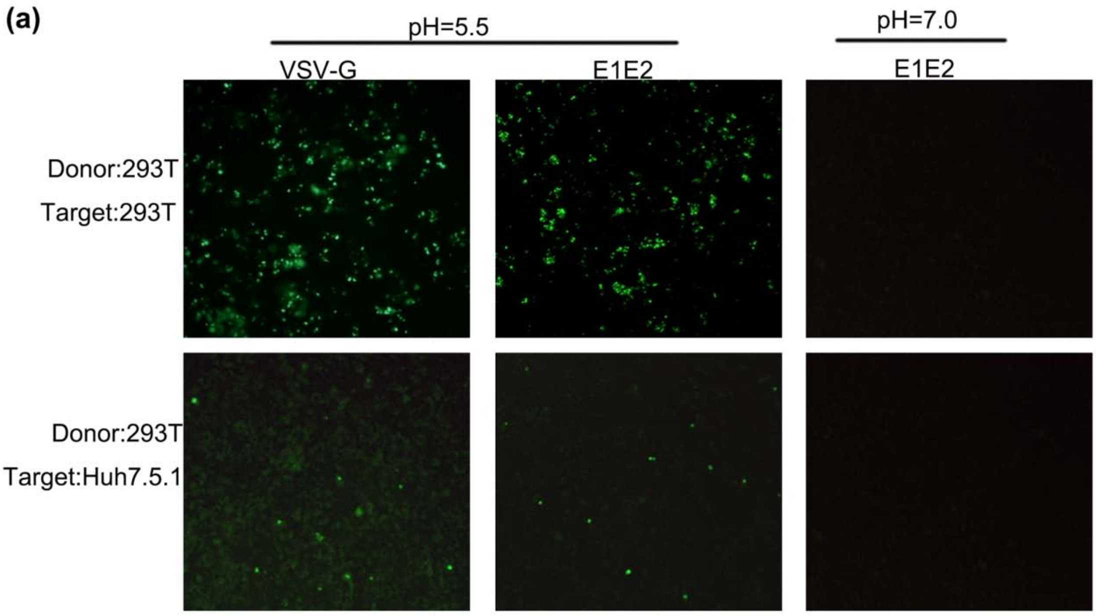

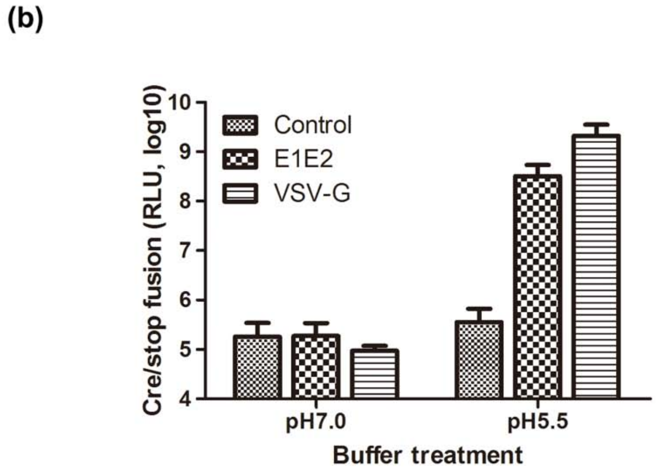

Supporting Figure S2.

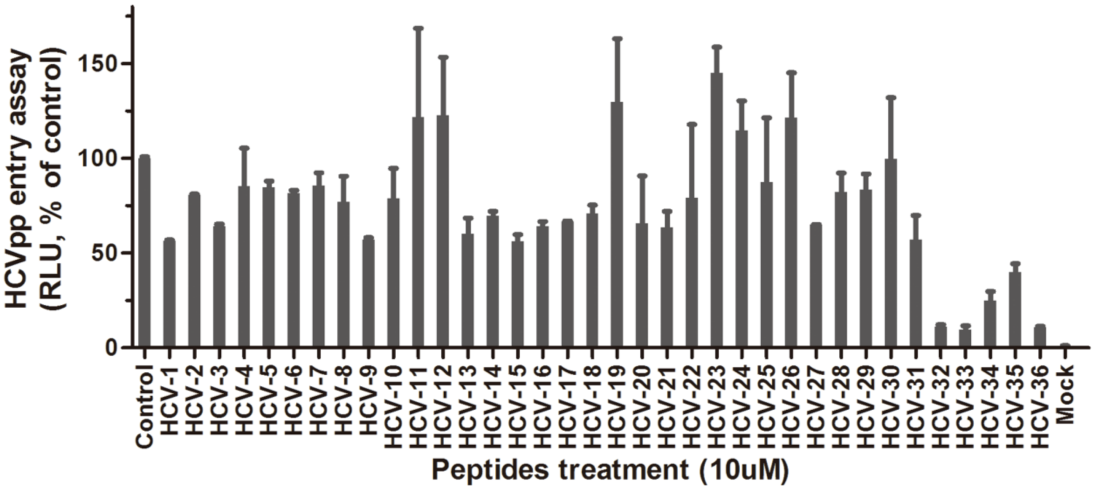

Supporting Figure S3.

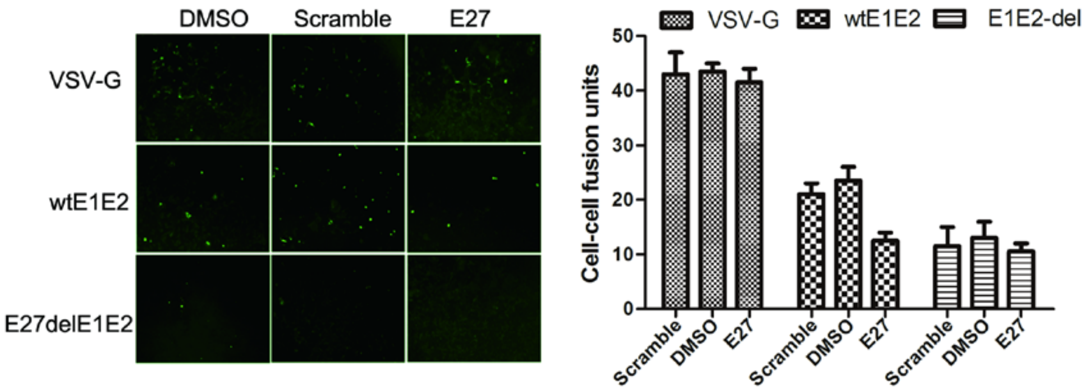

Supporting Figure S4.

(a)

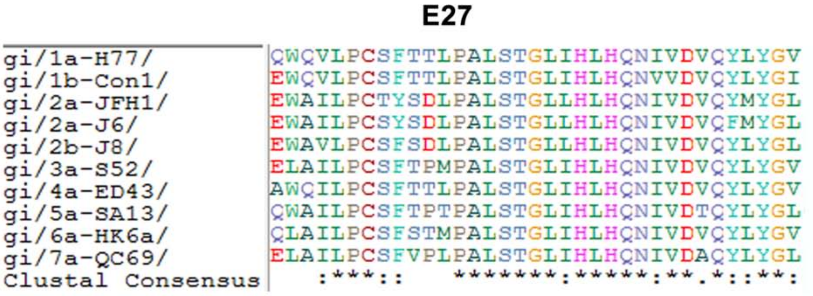

(b)

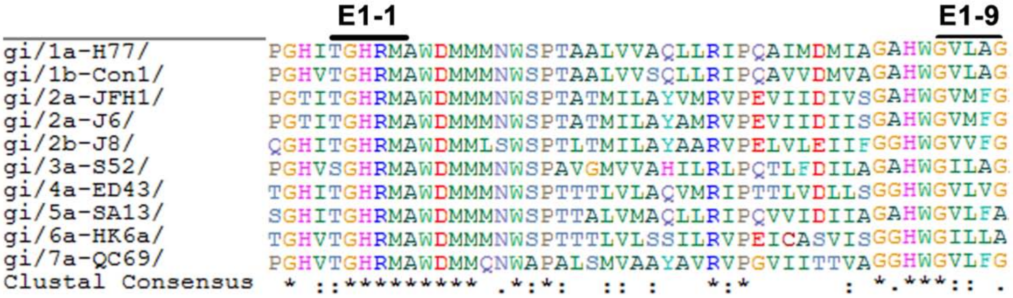

Supporting Figure S5.

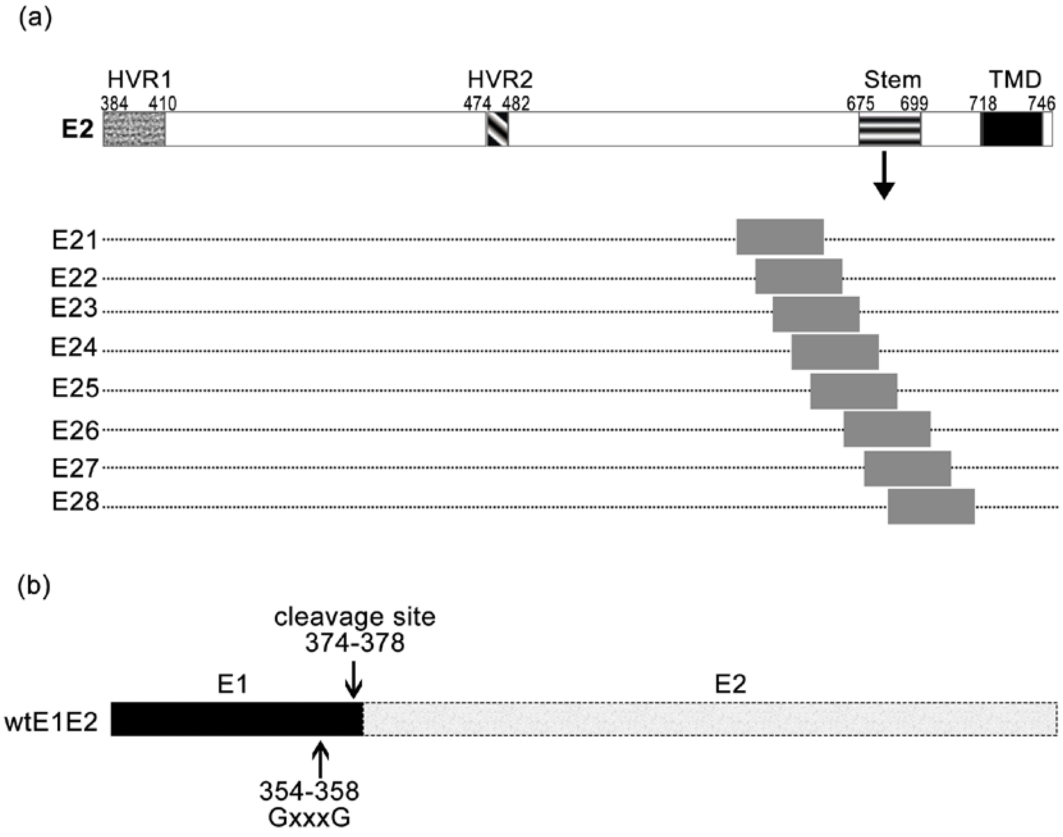

Supplement: Supplementary Information [file srep25224-s1.pdf]
